# A new model of cavern diameter based on a validated CFD study on stirring of a highly shear-thinning fluid

**DOI:** 10.1007/s11696-016-0119-y

**Published:** 2017-01-24

**Authors:** Anna Story, Zdzisław Jaworski

**Affiliations:** Institute of Chemical Engineering and Environmental Protection Processes, West Pomeranian University of Technology, Aleja Piastow 42, 71-065 Szczecin, Poland

**Keywords:** CFD, Validation, Shear-thinning, Stirring, Cavern, Energy dissipation

## Abstract

Results of numerical simulations of momentum transfer for a highly shear-thinning fluid (0.2% Carbopol) in a stirred tank equipped with a Prochem Maxflo T type impeller are presented. The simulation results were validated using LDA data and both tangential and axial force measurements in the laminar and early transitional flow range. A good agreement between the predicted and experimental results of the local fluid velocity components was found. From the predicted and experimental values of both tangential and axial forces, the power number, *Po*, and thrust number, *Th*, were also calculated. Values of the absolute relative deviations were below 4.0 and 10.5%, respectively, for *Po* and *Th*, which confirms a satisfactory agreement with experiments. An intensive mixing zone, known as cavern, was observed near the impeller. In this zone, the local values of fluid velocity, strain rate, Metzner–Otto coefficient, shear stress and intensity of energy dissipation were all characterized by strong variability. Based on the results of experimental study a new model using non-dimensional impeller force number was proposed to predict the cavern diameter. Comparative numerical simulations were also carried out for a Newtonian fluid (water) and their results were similarly well verified using LDA measurements, as well as experimental power number values.

## Introduction

Shear-thinning phenomenon is the most common deviation from the Newtonian behavior of fluids. Shear-thinning fluids, e.g. polymer solutions and melts, suspensions, emulsions and even food products are found in almost all industries. High shear forces occur near an impeller during stirring. With highly shear-thinning fluids in that region an intensive mixing zone called cavern (Wichterle and Wein 1975; Moore et al. 1995; Wilkens et al. 2005) is generated. Outside the cavern, the fluid velocity is close to zero. When stirring highly shear-thinning fluids, their rheological parameters have decisive influence on the intensive mixing zone size. Complex rheological properties of stirred fluids require proper selection of the agitator, system geometry and impeller rotational speed because the impeller parameters substantially affect efficiency of the mixing process.

In previous studies of caverns generated during mechanical stirring of highly shear-thinning fluids with or without yield stress either CFD numerical simulations (Bakker et al. 2009; Ein-Mozaffari and Upreti 2009; Pakzad et al. 2008; Saeed et al. 2007) or different experimental techniques were used. Among the latter, measurements of the fluid velocity were done using hot film anemometry (Solomon et al. 1981), Laser Doppler Anemometry, LDA, (Moore et al. 1995; Jaworski et al. 1994; Jaworski 2006), ultrasonic doppler velocimetry, UDV, (Ein-Mozaffari and Upreti, 2009; Hui et al. 2009; Saeed et al. 2007) or Particle Image Velocimetry, PIV, (Arratia et al. 2006; Gabelle et al. 2013). Other investigation methods include flow observation using tracer particles (Arratia et al. 2006), visualization using dye (Amanullah et al. 1997; Galindo and Nienow 1992), X-ray photography (Elson et al. 1986), electrical resistivity tomography, (Hui et al. 2009; Pakzad et al. 2008; Simmons et al. 2009), or even freezing (Wilkens et al. 2005). The studies were performed for different impellers, e.g. Rushton turbine (Arratia et al. 2006; Gabelle et al. 2013; Solomon et al. 1981), SCABA 3SHP1 or 6SRGT (Amanullah et al. 1997; Pakzad et al. 2008), Lightnin A315 or A310 (Ein-Mozaffari and Upreti 2009; Galindo and Nienow 1992; Saeed et al. 2007), Chemineer Maxflo (Hui et al. 2009), Pitched Blade Turbine (Ein-Mozaffari and Upreti 2009; Simmons et al. 2009; Solomon et al. 1981; Wilkens et al. 2005). The most commonly tested highly shear-thinning fluids were either transparent aqueous solutions of different concentrations of Carbopol (Amanullah et al. 1997; Arratia et al. 2006; Gabelle et al. 2013; Galindo and Nienow 1992; Simmons et al. 2009) or opaque Xanthan Gum (Ein-Mozaffari and Upreti 2009; Galindo and Nienow 1992; Solomon et al. 1981; Pakzad et al. 2008).

Authors of several publications proposed various empirical models to determine the diameter of caverns assuming their spherical, cylindrical or toroidal shape, based on the process operating parameters and rheological properties of stirred fluids. These models are usually suitable to predict cavern size of fluids with yield stress, *τ*_y_, in cases when a cavern diameter, *D*_c_, is less than a tank diameter, *T*, (Solomon et al. 1981, Eq. (1); Nienow and Elson 1988, Eq. (2); Elson et al. 1986, Eq. (2); Liu et al. 2010, Eq. (1); Galindo and Nienow 1992), e.g.:

1 $$\left( {\frac{{D_{\text{c}} }}{D}} \right)^{3} = \left( {\frac{4Po}{{\pi^{3} }}} \right)\left( {\frac{{\rho N^{2} D^{2} }}{{\tau_{\text{y}} }}} \right)$$

2 $$\left( {\frac{{D_{\text{c}} }}{D}} \right)^{3} = \left( {\frac{1.36Po}{{\pi^{2} }}} \right)\left( {\frac{{\rho N^{2} D^{2} }}{{\tau_{\text{y}} }}} \right)$$

where *D*_c_ is the cavern diameter, m, *D* is the impeller diameter, m, *Po* is the power number, *ρ* is the fluid density, (kg m^−3^), *N* is the impeller speed, s^−1^, *τ*_y_ is the yield stress, Pa. However, those models take into account tangential forces only. When mixing non-Newtonian, highly shear thinning fluids, the axial forces created by axial flow impellers have also an impact on the cavern size. The axial component of the momentum transferred locally by an impeller to the stirred fluid generates an impeller axial force. Therefore, the axial thrust of the impeller is also regarded as an important parameter.

Theoretical studies led to physical models incorporating the axial thrust of an impeller in the cavern size calculation (Amanullah et al. 1998; Bhole and Bennington 2010; Hui et al. 2009; Wilkens et al. 2005). Amanullah et al. (1998) proposed a new axial force model, Eq. (3), in which total momentum transferred to fluids is a sum of both tangential and axial force components, what is particularly important for impellers producing axial circulation inside stirred vessels:

3 $$\left( {\frac{{D_{\text{c}} }}{D}} \right)^{2} = \frac{1}{\pi }\sqrt {N_{\text{f}}^{2} + \left( {\frac{4Po}{3\pi }} \right)^{2} } \left( {\frac{{\rho N^{2} D^{2} }}{{\tau_{\text{y}} }}} \right)$$

where *N*_f_ is the dimensionless axial force number. Much less frequently, models were proposed for fluids obeying the Power Law without yield stress [Nienow and Elson 1988, Eq. (4); Amanullah et al. 1998, Eq. (5); Adams and Barigou 2007, Eq. (5)], e.g.:

4 $$\left( {\frac{{D_{\text{c}} }}{D}} \right)^{3} = \left( {\frac{{1.36k_{1} }}{{\pi^{2} k_{\text{s}} }}} \right)$$

5 $$r_{\text{c}}^{{\left[ {1 - \left( {2/n} \right)} \right]}} = v_{\text{o}} \left[ {\left( {\frac{2}{n} - 1} \right)\left( {\frac{4\pi K}{F}} \right)^{1/n} } \right] + b^{{\left[ {1 - \left( {2/n} \right)} \right]}}$$

where *k*_1_ is the laminar flow proportionality constant, *Po* = *k*_1_ *Re* ^−1^, *k*_s_ is the Metzner–Otto coefficient, *r*_c_ is the cavern radius, m, *v*_o_ is the fluid velocity at the cavern boundary, (m s^−1^), *n* is the flow behavior index, *K* is the fluid consistency coefficient, (Pa s^n^), *F* is the total force, N, *b* is the radius surrounding torus with a diameter equivalent to the vessel, m. The model (5) is based on the spherical cavern shape, with *r*_c_ = 0.5*D*_c_ and *b* = 0.5 *T*.

A very similar model (Amanullah et al. 1998; Adams and Barigou 2007) was created assuming toroidal shape of the cavern, by substituting the *π* value by *π* ^2^ and *b* = 0.5 *T* by *b*_t_ = 0.25 *T*. In that model the calculated value of cavern radius is *r*_c,t_ = 0.25*D*_c_.

The purpose of this work was to determine the impact of the rotational speed of a Prochem Maxflo T (PMT) type impeller, with pitched and profiled blades, onto the hydrodynamics of a highly shear-thinning fluid, characterized by high values of apparent viscosity. LDA studies for that system were published by Jaworski and Nienow (1994) and Jaworski (2006). The former authors presented results of Laser Doppler Anemometry measurements within a cavern for all 3 velocity components at one rotational impeller speed (*N* = 8 s^−1^). However, data of the tangential velocity component were collected for a wide-range of the impeller speed, *N* = 2–12 s^−1^ and only at the impeller center level. Therefore, in the present paper, the CFD numerical simulations are also presented for that impeller speed range.

To verify results of the numerical simulations, values of the three CFD velocity components were compared with available experimental values from ensemble-averaged mode LDA measurements (Jaworski and Nienow 1994). In addition, measurements of the total tangential and axial forces in the tank were carried out enabling calculation of the power number and the thrust number. Then the experimental total force values were used to compare with analogous data obtained from own CFD modeling.

Based on the simulation results, the local values of the strain rate, $\dot{\gamma }$, Metzner–Otto coefficient, *k*_sℓ_ (Eq. (18)), shear stress, *τ*_ℓ_ (Eq. (6)), and intensity of energy dissipation, *ε*_ℓ_ (Eq. (19)), were calculated for the studied impeller speed range and presented as distribution contours. Predictions of the cavern diameter based on the numerical simulations and literature models were also compared. Finally, a new model for cavern diameter in a system with PMT type impeller was proposed.

To compare the hydrodynamic conditions created by the PMT type impeller in the Carbopol solution with those for water as a Newtonian fluid, numerical simulation for water were also carried out. Then the CFD results were presented as velocity vectors and compared with the corresponding LDA results for 0.2% Carbopol solution and water. Results of the simulations were favorably verified using the LDA measurements and power number experimental values from the literature (Jaworski et al. 1996) and also new measurements of both the tangential and axial forces.

## The studied system

**Fig. 1:**
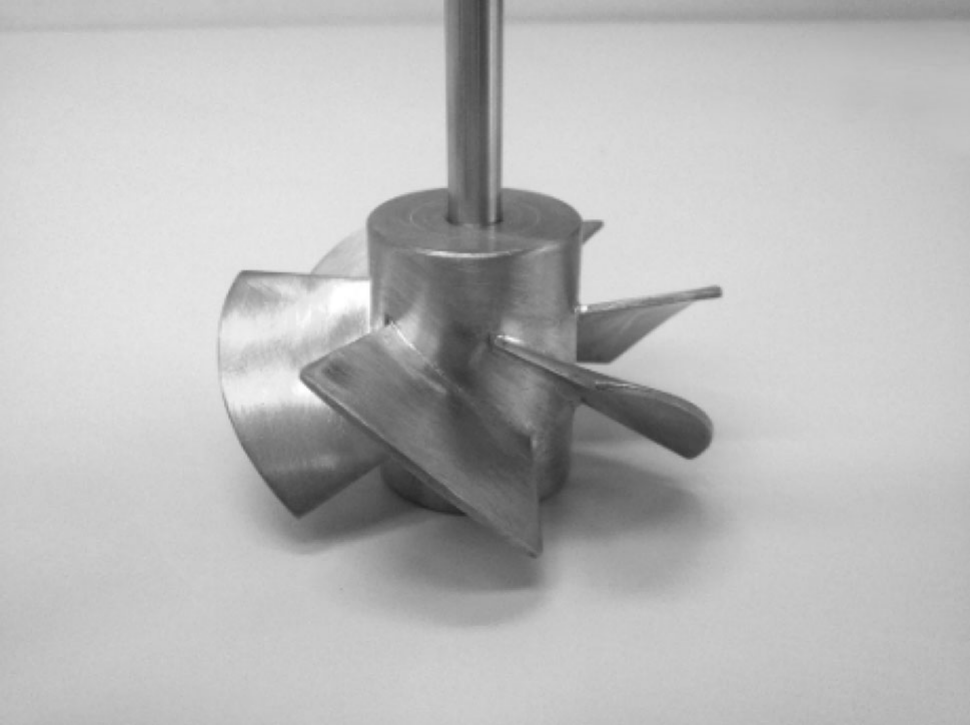
PMT type impeller

The CFD numerical simulations were performed for a flat-bottomed stirred tank equipped with four flat, standard baffles and a PMT type impeller with a geometry analogous to the Prochem Maxflo T (Jaworski and Nienow 1994) (Fig. 1). Basic parameters of the studied system are presented in Table 1.

**Table 1:** Basic data of the stirred tank

Tank	
Diameter, *T*/m	0.222
Liquid high, *H*	*T*
Number of baffles	4
Width of baffle, *w*	0.1 *T*
Impeller	
Diameter, *D*/m	0.078
Number of blades	6
Clearance	0.45 *H*
Rotational speed, *N*/s^−1^	2, 4 (4.15)^a^, 6, 8, 10, 12

^a^Value for water

**Fig. 2:**
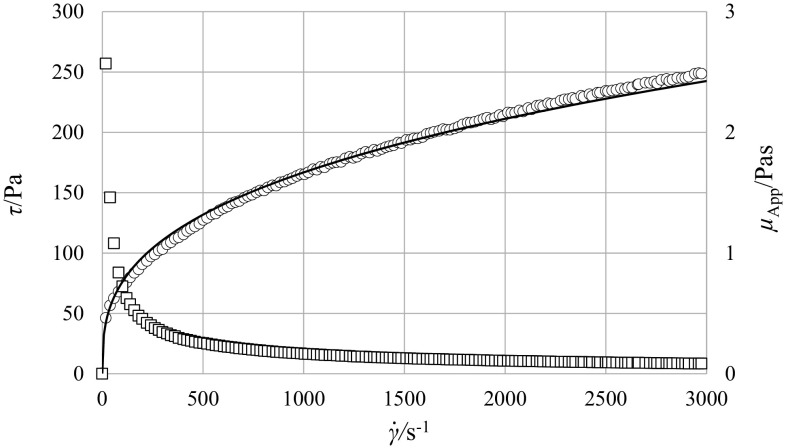
Rheological properties of the 0.2% aqueous Carbopol solution [apparent viscosity (*square*), shear stress (*circle*) and model $\tau = 15.9 \cdot \dot{\gamma }^{0.34}$ (*line*)]

The two tested fluids were water and an aqueous solution of Carbopol (Lubrizol, 940 grade) of 0.2% wt. concentration and pH ≈5.1. Rheological properties of the Carbopol solution were measured using the Rotovisco RT 10 rheometer (Haake). Temperature of the fluid was kept at a constant level of 20.5 ± 0.5 °C. Experimental values of the apparent viscosity, *µ*_App_, and shear stress, *τ*, were presented as a function of the strain (shear) rate, $\dot{\gamma }$, in Fig. 2. It follows from the figure, the fluid satisfactorily obeyed the rheological Power Law in a wide-range of shear rate, and its rheological characteristic was presented as:

6 $$\tau = K \cdot \dot{\gamma }^{n}$$

where the consistency coefficient, *K*, was equal to 15.9 Pa s^n^ and the flow behavior index, *n*, was much less than 1 and equal to 0.34. Such a low value of *n* corresponds to highly shear-thinning fluids.

## Numerical simulations

Basic to the momentum transfer modeling are in computational fluid dynamics (CFD) the differential balance equations for momentum transport (7) and continuity (8), which were solved in every computational cell assuming continuity of the liquid and its constant density.

7 $$\frac{{\partial {\mathbf{v}}}}{\partial t} + {\text{div }}\left( {{\mathbf{vv}}} \right) = {\text{div }}\left( {\nu \,{\text{grad}}\, {\mathbf{v}}} \right) - \frac{1}{\rho }{\text{div}} \left( {p{\mathbf{I}}} \right) + {\mathbf{g}}$$

8 $${\text{div }}{\mathbf{v}} = 0.$$

Geometry and mesh of the system were created using ANSYS DesignModeler and ANSYS Meshing software, respectively. Computational cells were generated by means of a numerical grid applied to the stirred liquid volume. The liquid volume was divided into about 798 thousand tetrahedral computational cells. The Wall boundary condition was applied for the tank wall and bottom, baffles, impeller and shaft along with the symmetry boundary condition for the free surface of the stirred fluid to mimic the zero shear stress there. The simulations were performed by means of the commercial ANSYS Fluent 14.5 software. The transport equations were discretized using the finite volume method and next solved using the second order upwind scheme. The simulations were carried out for the laminar and early transitional flows of Carbopol using the laminar model alone or with one of the two turbulence models for low values of the Reynolds number: *k*–*ɛ* or *k*–*ω*. In the case of non-Newtonian fluid flow modeling, a rheological equation of the fluid should be added to the standard system of equations. In the studied non-Newtonian case, the viscosity, *μ*, in Eq. (7) was replaced by the apparent viscosity, *μ*_App_, which for the incompressible, power law fluids can be calculated from equation:

9 $$\mu_{\text{App}} = \frac{\tau }{{\dot{\gamma }}} = K \cdot \dot{\gamma }^{{\left( {n - 1} \right)}} .$$

In the applied CFD code the minimum and maximum acceptable values of viscosity were also defined to aid the simulations. They amounted to *μ*_min_ = 1.003 × 10^−3^ Pa s and *μ*_max_ = 10 Pa s, respectively.

The numerical calculations were of two-stage character. In the first stage, they were performed in the steady-state mode using the multiple reference frame (MRF) method. After reaching convergence of the iterations, i.e. a minimum of residuals, the simulations were continued in the transient mode using the sliding mesh (SM) method, with a very small time step. Correctness of the time step selection was verified by calculating values of the convection Courant number (Ferziger and Perić 1999):

10 $$C_{\text{k}} = \frac{v \cdot \Delta t}{\Delta x}$$

where *v* is the local fluid velocity, Δ*t* is the time step, Δ*x* is the spatial step. To ensure stability of the calculations, values of the Courant number for the upwind scheme should satisfy the condition *C*_k_ ≤1 (Tannehill et al. 1997). The impeller peripheral velocity, *v*_TIP_ = *πDN*, was adopted as the maximum fluid velocity. Preliminary simulations were performed and allowed to select an optimal time step, corresponding to the impeller rotation by an angle of 2°. For this angle, the maximum Courant number, *C*_k,max_, for fluid volume inside the impeller region was equal to 1, so the condition *C*_k_ ≤1 was satisfied. Outside the volume, the fluid velocities were lower than *v*_TIP_, consequently *C*_k_ <1. The decisive criterion for completing the iteration process within one time step was to achieve a minimum—plateau of the numerical residues sum. For one time step, the internal iterations number amounted up to 100, while the number of time steps was 600.

To validate the CFD procedure the final numerical results, in form of the three velocity components and both the tangential and axial stresses, were compared with corresponding results from LDA, experimental power number and thrust number data. Then, for the non-Newtonian fluid, local values of shear rate for every computational cell were read and from them the local values of the Metzner–Otto coefficient, shear stress and intensity of energy dissipation were also calculated.

## Torque and thrust measurements

Power number in mechanically stirred tanks can be derived either by integration of mechanical energy dissipated in the tank volume per time unit, *P*, or on the basis of the transferred energy resulting from torque, *T*_o_, exerted by the impeller and shaft (primary torque) or from torque acting on the tank wall, bottom and baffles (secondary torque).

In this study, the second method was used and values of the primary torque were measured using the IKA^®^ EUROSTAR Power Control-Visc device. Measurements of the torque were realized for impeller speeds ranging from 0.83 to 13.33 s^−1^. For every impeller speed, 40 instantaneous values of the torque were read at equal time intervals (every 5 s), and averaged. Then, the mixing power, *P*, and power number, *Po*, were calculated from:

11 $$P = 2\pi NT_{\text{o}}$$

12 $$Po = \frac{P}{{N^{3} D^{5} \rho }}.$$

Experimental values of the power number, *Po*_exp_, were then used to verify the power numbers obtained from simulations, *Po*_CFD_.

The axial thrust of axial flow impellers can be determined in two ways (Fort et al. 2013): by experimental measurements of the total axial force, *f*_ax_, or by calculation of the axial momentum affecting the impeller on the basis of the axial component of the mean velocity. In this paper, the first way was used in measurements, and the axial thrust was determined by directly measuring the change in weight of the tank with the stirred charge. The second way of *f*_ax_ determination was applied in the CFD simulations.

**Fig. 3:**
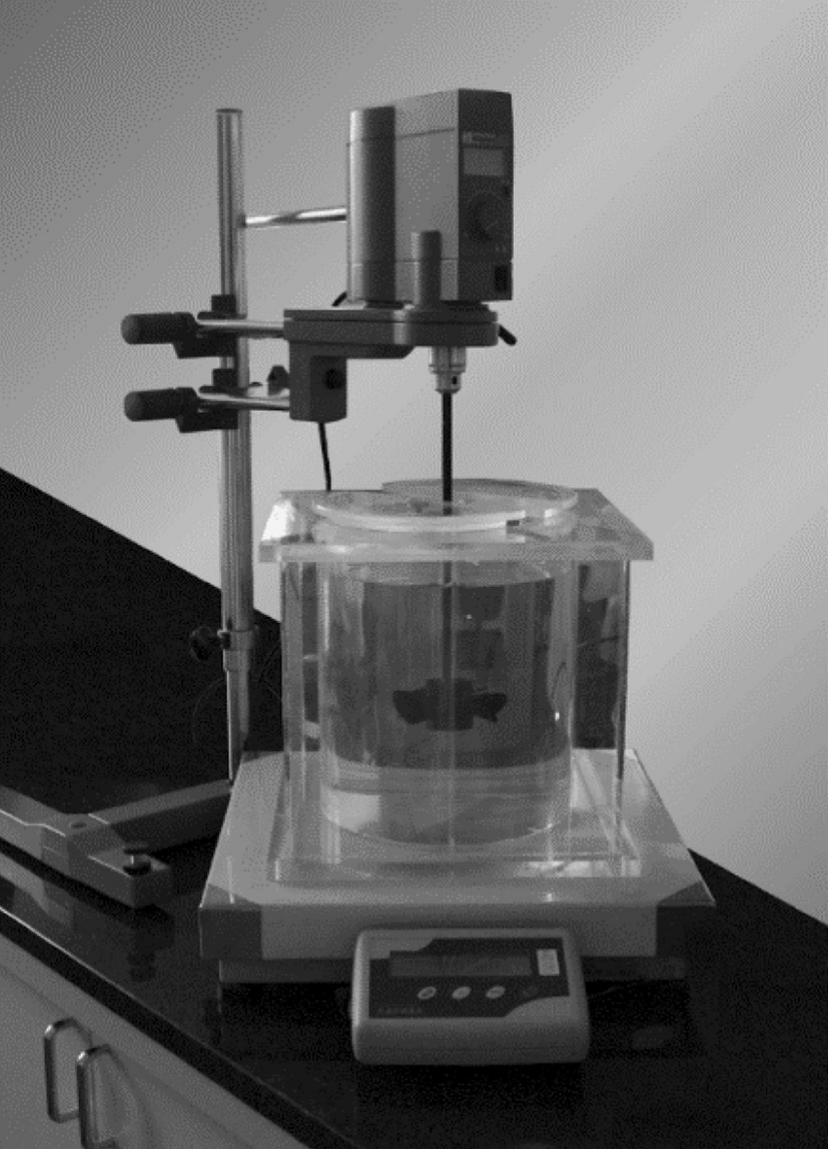
Experimental system

In the experiments, see Fig. 3, the mixing tank was placed on electronic WPT 24C Radwag scales of the maximum load of 24 kg and measuring accuracy of 1 g. Measurements of the total axial force were realized for the same impeller speeds as in the torque measurements. As before, for every impeller speed, 40 instantaneous values of the weight (mass) were read at equal time intervals (every 5 s). Then the instantaneous values were reduced by the weight of the system without agitator rotation, and averaged. Multiplying the average value of the reduced mass by acceleration due to gravity, mean values of the force acting in the axial direction (axial thrust), *f*_*ax*_, were obtained. Subsequently, on the basis of the axial thrust of the impeller, the dimensionless thrust number, *Th*, was calculated from equation (Fort et al. 2013):

13 $$Th = \frac{{f_{\text{ax}} }}{{\rho N^{2} D^{4} }}.$$

## Cavern size measurements

For cavern visualization, a planar laser-induced fluorescence was used. First, a laser beam from green laser with an output power 50 mW was formed into a sheet and then it was passing vertically through the system in a mid-plane located between adjacent baffles. Images revealing size of the cavern were captured by the camera Canon EOS 1200D which was placed at right angle to the laser sheet. For observing caverns, a passive dye tracer—Rhodamine 6G solution, was injected into the mixing fluid near the impeller hub, between the impeller blades. The solution was prepared by dissolving 5 mg/l of the powdered dye in the tested fluid, 0.2% Carbopol. The addition of the dye solution did not change measurably the rheological properties of the tested Carbopol solution, what was confirmed in series of the torque measurements. After adding 20 ml of the dye solution into the fluid, the PMT type impeller rotated at the lowest constant speed until stabilization of the cavern size was reached. Then an image of the system was taken. Analogous measurements were repeated for increased impeller speeds. However, before increasing the impeller speed, the drive was always stopped to add another portion of the dye (10 ml) to the system. Due to symmetry of the system, the results are graphically presented only for a half of the tank.

## Validation

### Mean velocity

Numerical simulations performed for 0.2% Carbopol with the sliding mesh option allowed to obtain local, instantaneous values of the three velocity components: axial, *v*_z,CFD_, radial, *v*_r,CFD_, and tangential, *v*_t,CFD_. After reaching stable convergence at consecutive 30 time steps, local values of the instantaneous velocity in a vertical plane located at the angle of 45° between baffles were stored. The points of data reading were located similarly to those in the experiment (Jaworski and Nienow 1994), i.e. in a vertical mid-plane between neighboring baffles and along horizontal lines in nine axial positions, *z* = −40, −30, −20, −10, 0, 10, 20, 30, 40 mm; where *z* = 0 corresponded to the axial coordinate of the impeller center. Next, for each point, 30 consecutive values of the instantaneous velocity were averaged to obtain the mean local values of the velocity components, $\bar{v}_{{i , {\text{CFD}}}}$, where *i* = *z*, *r*, *t*. That averaging corresponded to the impeller rotation by an angle of 60° equal to the angular distance between two adjacent impeller blades. Dimensionless velocity components, $V_{{I , {\text{CFD}}}}$, were obtained by dividing local values of the mean velocity components, $\bar{v}_{{i , {\text{CFD}}}}$, by the peripheral impeller tip speed (14), whereas dimensionless radial distances, *R*, were calculated by dividing radial distances off the tank axis to the reading points, *r*, by the tank diameter, *T*, (15):

14 $$V_{{I , {\text{CFD}}}} = \frac{{\bar{v}_{{i , {\text{CFD}}}} }}{\pi DN} \, \left( {i = z, \, r, \, t; \, I = Z, \, R, \, T} \right)$$

15 $$R = \frac{r}{T}.$$

Profiles of the three dimensionless mean velocity components were subsequently plotted as a function of the dimensionless radial distance.

**Fig. 4:**
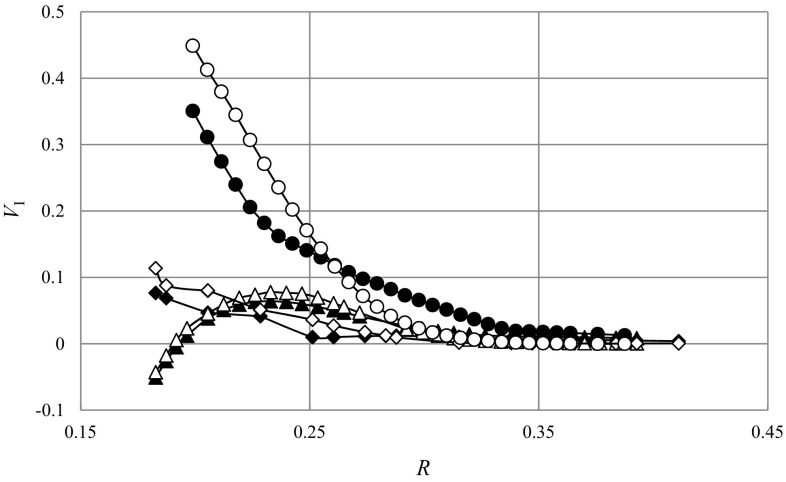
Radial profiles of the three mean dimensionless velocity components along dimensionless radius at height *z* = 0 mm, *N* = 8 s^−1^ [CFD, axial velocity (*filled triangle*); CFD, radial velocity (*filled rhombus*); CFD, tangential velocity (*filled circle*); LDA, axial velocity (un*filled triangle*); LDA, radial velocity (un*filled rhombus*); LDA, tangential velocity (*filled circle*); LDA data from Jaworski and Nienow (1994)]

Sample profiles of the local mean dimensionless velocities for the height *z* = 0 mm, obtained from numerical simulations, $V_{{I , {\text{CFD}}}}$, and from LDA measurements, $V_{{I,{\text{LDA}}}}$, are shown in Fig. 4. Analyzing the results, it was found that intensive fluid mixing illustrated by high values of the velocity components, occurred only near the impeller and created a typical cavern. Furthermore, outside the impeller swept volume and inside the cavern, maximum values of velocity were noted for the tangential component and they were over two times smaller than the impeller tip speed, cf. Figure 4. At the cavern boundary all three velocity components had low values, while outside the cavern they were very close to zero.

**Fig. 5:**
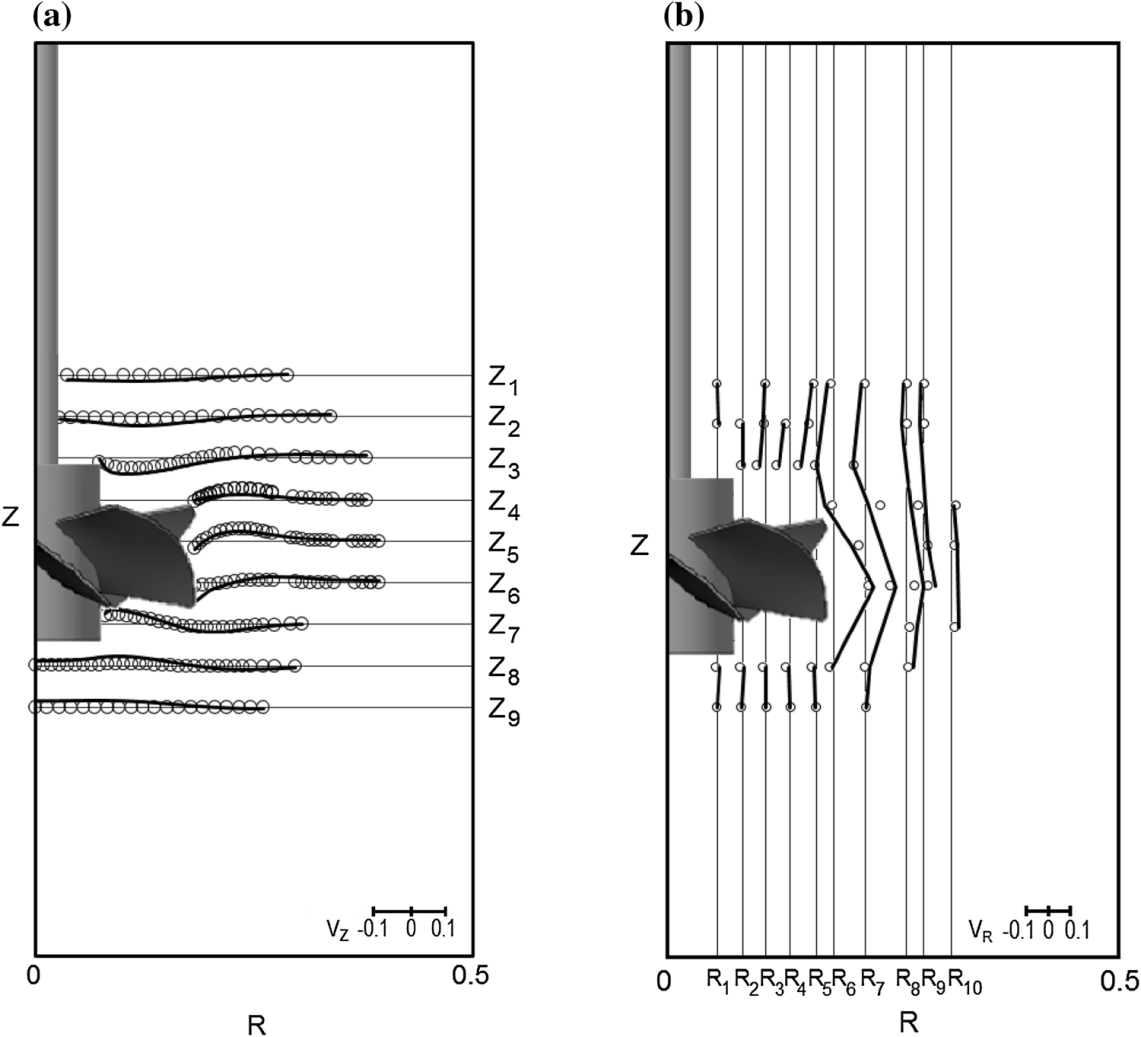
Profiles of the dimensionless velocity components for *N* = 8 s^−1^ in Carbopol: **a** radial profiles of the dimensionless axial mean velocity component, *V*_Z_, along dimensionless radius, **b** axial profiles of the dimensionless radial mean velocity component, *V*_R_, along dimensionless height [CFD (*line*), LDA (*circle*), LDA data from Jaworski et al. (1996)]

Figure 5 shows a graphical comparison between the predicted (lines) and experimental (points) velocity values for the aqueous Carbopol solution in the form of radial profiles of the dimensionless axial mean velocity component along the dimensionless radius at different heights (Fig. 5a) and in the form of axial profiles of the dimensionless radial mean velocity component along the height at different radial positions (Fig. 5b). The data were read in a plane located at the angle of 45° between baffles for impeller speed *N* = 8 s^−1^. The solid lines represent values obtained from numerical modeling while circles represent literature data from LDA measurements (Jaworski and Nienow 1994).

Subsequently, the results shown in Fig. 5 and also those for the tangential velocity component obtained from CFD for the impeller speed *N* = 8 s^−1^ were compared quantitatively with the analogous LDA results (Jaworski and Nienow 1994), and the mean square deviations between corresponding local dimensionless velocities were determined from:

16 $$\sigma = \sqrt {\frac{{\sum {\left( {V_{{I,{\text{LDA}},j}} - V_{{I,{\text{CFD}},j}} } \right)^{2} } }}{{\left( {n - 1} \right)}}} \times 100\% \quad{\text{ for }}j = 1 \ldots n.$$

In the case of the laminar model used for Carbopol solution, the deviation values of 2.1, 2.5 and 3.6% of *v*_TIP_ were obtained for the axial (*n* = 223), radial (*n* = 210) and tangential (*n* = 198) velocity components, respectively, which proves a good agreement between the predicted and experimental results. Slightly worse accordance of the predicted and experimental results was observed when one of the turbulence models was enabled. In the case of the Low *Re k*–*ɛ* model, the deviation values were found close to 2.9, 3.9 and 7.3% of *v*_TIP_ for the axial, radial and tangential velocity components, respectively, while in the case of the Low *Re k*–*ω* model the same velocity components differed by 2.5, 3.7 and 4.9% of *v*_TIP_. The discrepancies were relatively high in the vicinity of the impeller blades. In view of the worsening results for enabled turbulence models, the simulations for other impeller speeds were performed using the laminar model only.

The differences in values of the three mean velocity components obtained from the CFD simulations and LDA measurements can result from different ways of the local data reading and averaging. In the case of numerical simulations, the local values were averaged from 30 instantaneous values stored at selected points for consecutive 30 time steps. However, an LDA velocity measurement is taken when a tracer particle flows across the volume formed by two crossed laser beams at the time. The compared components of the local velocity were calculated in LDA as ensemble averages from hundreds of instantaneous values at a chosen point for different angular positions of the impeller blades. It should also be mentioned that in the present work the 0.2% Carbopol solution was treated as a non-Newtonian highly shear thinning fluid, on the basis of literature data. However, this polymer containing fluid may have slightly elastic properties, which could also affect the differences between predicted and experimental data, especially close to the impeller blades where shear rates are high.

**Fig. 6:**
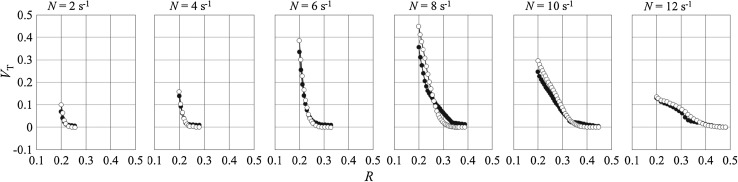
Radial profiles of the dimensionless tangential mean velocity component along dimensionless radius at height *z* = 0 mm, *N* = 2–12 s^−1^ [CFD (*filled circle*), LDA (*unfilled circle*), LDA data (Jaworski and Nienow unpublished work)]

Figure 6 shows averaged dimensionless values of the dominant tangential velocity component for different impeller rotational speeds. For instance, this velocity component attained values close to zero for the radial distance of about *R* = 0.22 and *R* = 0.43 for the impeller rotation speed *N* = 2 s^−1^ and *N* = 12 s^−1^, respectively. Increase of the distance from the tank axis to the point where the tangential velocity component becomes close to zero with growing impeller speed correlates with simultaneous growth of the intensive mixing zone (cavern).

**Fig. 7:**
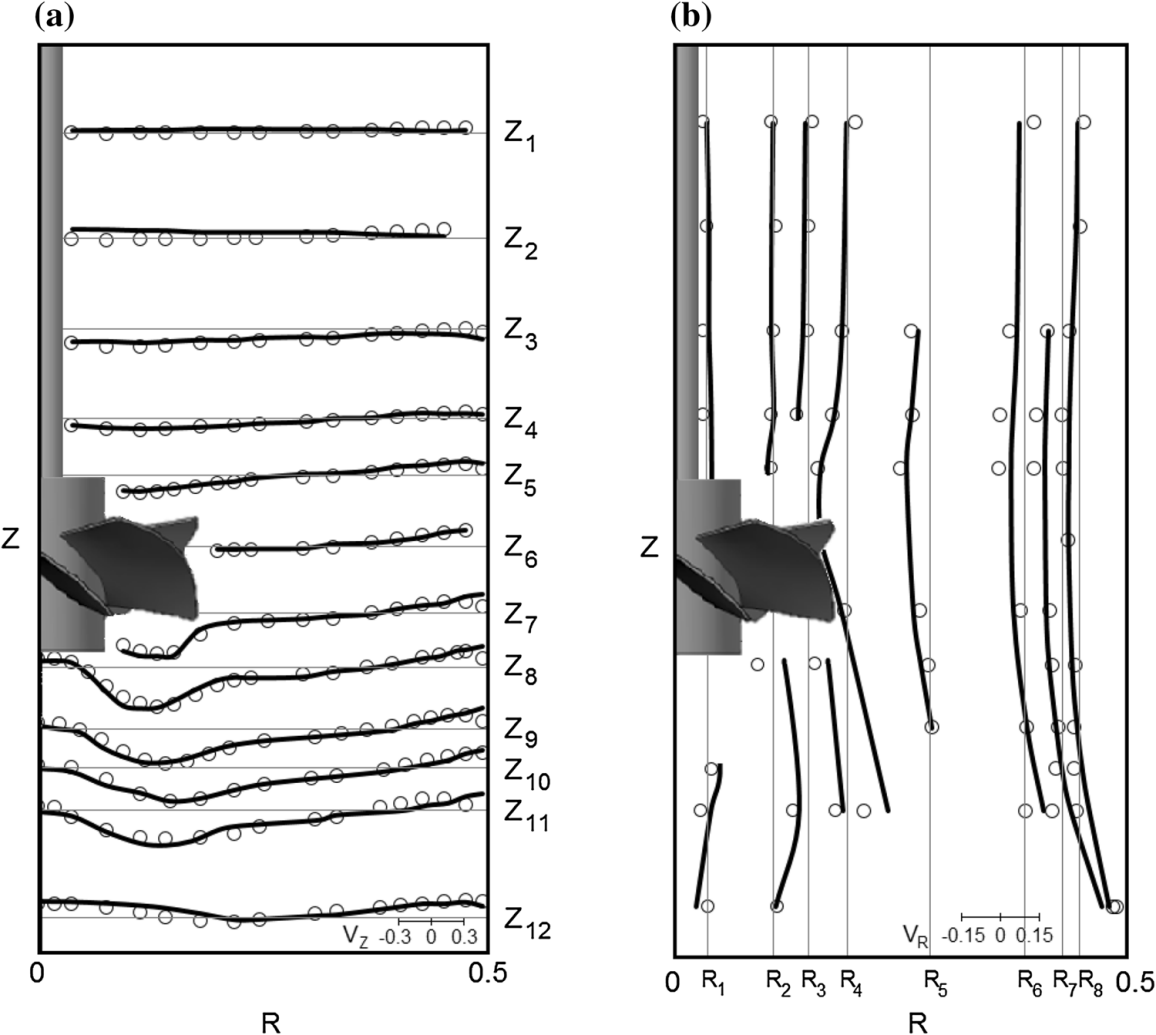
Profiles of the dimensionless velocity components for *N* = 4.15 s^−1^ in water: **a** radial profiles of the dimensionless axial mean velocity component, *V*_Z_, along dimensionless radius, **b** axial profiles of the dimensionless radial mean velocity component, *V*_R_, along dimensionless height [CFD (*line*), LDA (*circle*), LDA data from Jaworski et al. (1996)]

In the case of the Newtonian fluid (water) the CFD simulations had also the two-stage character, however the modeling was carried out with the *k*–*ɛ* turbulence model and standard wall functions enabled. The same procedure of data reading and averaging as for the non-Newtonian fluid was applied. Radial and axial profiles of both axial and radial dimensionless velocity components are plotted in Fig. 7 to qualitatively compare results obtained from CFD and LDA (Jaworski et al. 1996). A quantitative comparison was also performed. Calculated values of the mean square deviations (Eq. 16) between the predicted and experimental data were 5.0, 3.9, 1.9% of *v*_TIP_ for the axial, radial and tangential velocity components, respectively.

### Power number

In this study, the method based on the primary torque derived from CFD was used. Values of the primary torque, *T*_o_, were read directly in the ANSYS Fluent software from simulation results. The mixing power, *P*, and power number, *Po*, were calculated from Eqs. (11) and (12).

Values of the percentage absolute relative deviation between the experimental and predicted values were calculated from equation:

17 $$\delta = \frac{{\left| {Po_{\exp } - Po_{\text{CFD}} } \right|}}{{Po_{\exp } }} \times 100\% .$$

**Fig. 8:**
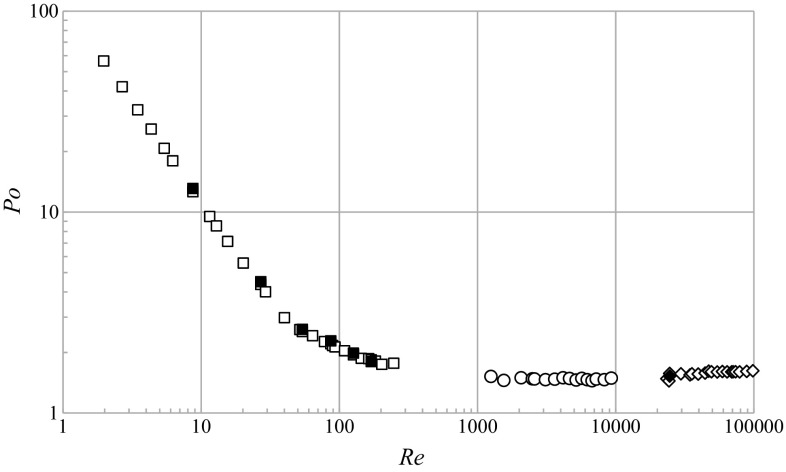
Mixing power characteristic [0.2% Carbopol, CFD (*filled square*); water, CFD (*filled rhombus*); 0.2% Carbopol, experiment (*unfilled square*); water, experiment (*unfilled rhombus*); glucose syrup, experiment (*unfilled circle*)]

The power number values calculated from the numerical results and experimental data for impeller speeds from 2 to 12 s^−1^ are summarized in Table 2. The maximum value of absolute relative deviation for *Po* was equal to 3.7%, which proves a satisfactory agreement between the compared results. The power number data were also supplemented with corresponding values of the Reynolds number, $Re_{\text{CFD}} = ND^{2} {\rho \mathord{\left/ {\vphantom {\rho {\left( {K \cdot \bar{\dot{\gamma }}_{\text{CFD}}^{{\left( {n - 1} \right)}} } \right)}}} \right. \kern-0pt} {\left( {K \cdot \bar{\dot{\gamma }}_{\text{CFD}}^{{\left( {n - 1} \right)}} } \right)}}$. Mean values of the strain rate in numerical simulations, $\bar{\dot{\gamma }}_{\text{CFD}}$, were calculated on the basis of the Metzner–Otto method, which is described in the next section. Next, the mixing power characteristic is plotted as shown in Fig. 8.

**Table 2:** Experimental and predicted values of the power number, total axial force and thrust number for different Reynolds numbers in 0.2% Carbopol

*Re*	*Po*_exp_	*Po*_CFD_	*δ*_*Po*_, %	*f*_ax,exp_, N	*f*_ax,CFD_, N	*Th*_exp_	*Th*_CFD_	*δ*_*Th*_, %
9	12.61	13.08	3.7	0.228	0.211	1.52	1.43	5.9
27	4.36	4.50	3.2	0.360	0.328	0.61	0.55	9.7
54	2.54	2.60	2.4	0.491	0.475	0.37	0.36	3.2
87	2.21	2.28	3.2	1.272	1.143	0.54	0.48	9.8
126	1.95	1.98	1.5	2.238	2.046	0.60	0.55	8.0
170	1.84	1.80	2.2	3.364	2.997	0.63	0.56	10.4

Analogous calculations were carried out for water giving values of the experimental, *Po*_exp_ = 1.57 and predicted, *Po*_CFD_ = 1.53, thus their absolute relative deviation was 2.5%. Figure 8 also shows the experimental power data for another Newtonian fluid, an aqueous solution of glucose syrup, having a viscosity of about 12 mPa s.

### Axial thrust

**Fig. 9:**
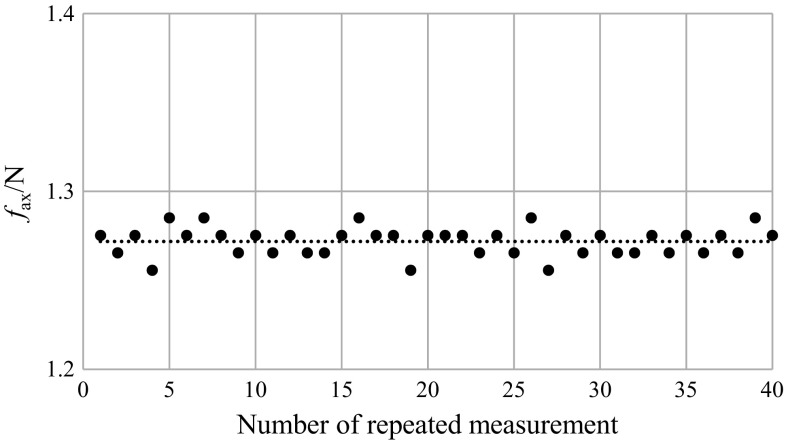
Fluctuation of the experimental axial force, *N* = 8 s^−1^ [instantaneous values (*filled circle*), mean value $\bar{f}_{\text{ax}} = 1.27{\text{ N}}$ (*dotted lines*)]

**Fig. 10:**
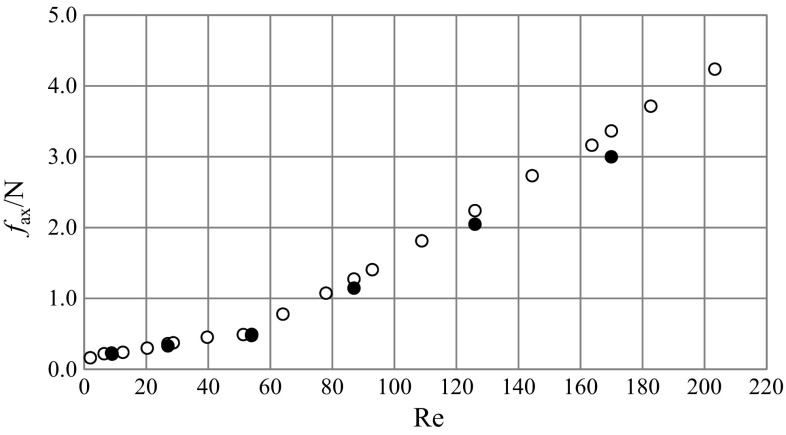
Total axial force for different *Re* numbers [CFD (*filled circle*), experiment (*unfilled circle*)]

**Fig. 11:**
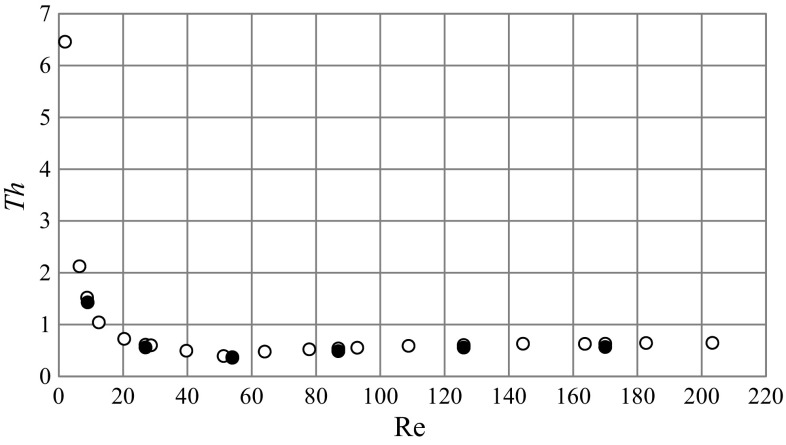
Dimensionless thrust number for different *Re* numbers [CFD (*filled circle*), experiment (*unfilled circle*)]

Values of the total axial force, *f*_ax_, induced by the rotating agitator were directly read from the numerical simulation results. Then from Eq. (13), values of the predicted *Th*_CFD_ number for different impeller speeds were calculated and compared with experimental data. The results are presented graphically in Figs. 9, 10 and 11.

Figure 9 shows exemplary variations within ±1.3% in the experimental instantaneous value of the force acting in the axial direction for the selected rotational impeller speed, *N* = 8 s^−1^.

Changes in the mean values of the total axial force, *f*_ax_, for various Reynolds numbers are shown in Fig. 10. Axial force values obtained from the experiment and from CFD are both presented in the graph. It was found that with increasing impeller speed the total axial force also increased. Changes of the *f*_ax_ were minor for *Re* <50, while for *Re* >50, the increase was rapid.

Figure 11 presents values of the dimensionless thrust number calculated from Eq. (13) on the basis of experiments and CFD simulations for different Reynolds numbers. It was observed that with increasing *Re* numbers values of the *Th* number initially strongly decreased, from about *Th* = 6.5 for *Re* = 2, reaching a minimum value (*Th* = 0.37) for *Re* = 54, then increased and oscillated within *Th* = 0.52–0.64. Table 2 also contains numerical values of *f*_ax_ and *Th* obtained from the experiment and numerical modeling for the studied impeller speed range, *N* = 2–12 s^−1^. It was also concluded, that the CFD predicted values of *f*_ax_ and *Th* were close to those obtained from experiments. Values of their percentage absolute relative deviation, *δ*_Th_, are also included in Table 2.

From comparison of the CFD simulation results with experimental data for all the analyzed quantities: the three velocity components, power number and thrust number, one can conclude that always a satisfactory agreement was obtained. Thus, it can be safely assumed that the applied modeling procedure was correct and its results can be used in further analysis of flow patterns and cavern size.

## Discussion of results

### Flow patterns

**Fig. 12:**
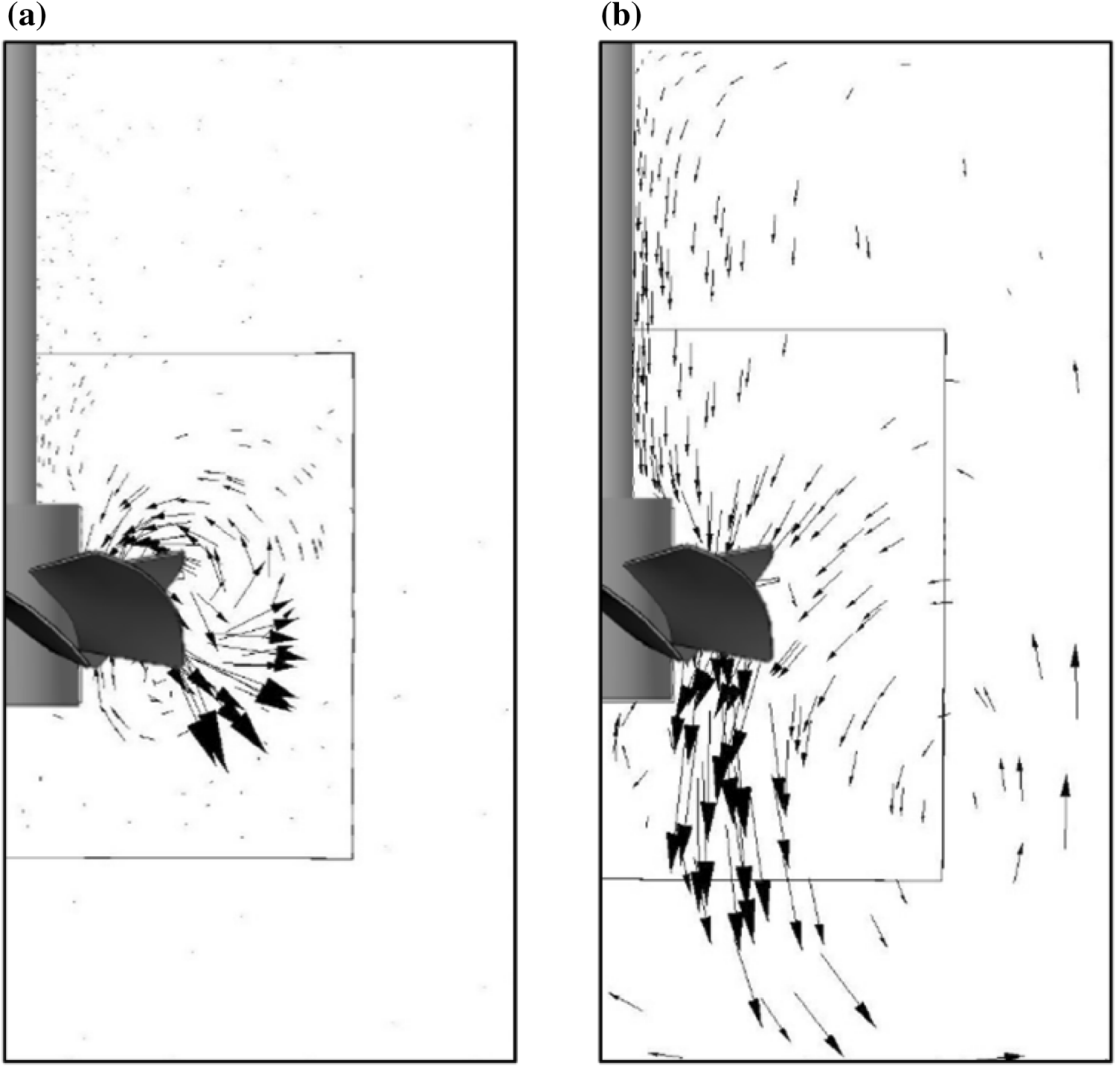
Velocity vectors in a mid-plane located between adjacent baffles: **a** 0.2% Carbopol, *N* = 8 s^−1^, **b** water, *N* = 4.15 s^−1^

Typical results of numerical simulations for both non-Newtonian and Newtonian fluids are presented in Fig. 12 as velocity vectors in a vertical plane located at the angle of 45° between adjacent baffles. Analyzing the vectors for Carbopol solution it was found that for the impeller speed *N* = 2 s^−1^, the fluid was effectively stirred only in the close proximity to the impeller blades, and the size of the primary circulation loop increased with increasing impeller speed up to *N* = 12 s^−1^.

As expected, the hydrodynamic conditions for water were quite different. It was found that the primary circulation loop spreads from the impeller to the bottom of the tank occupying a major part of the tank. There were also two zones of secondary flow. A small one was underneath the impeller hub, the second in the top area of the tank at the free surface of the stirred water. The flow intensity of the secondary circulation loops was much lower than that in the primary loop.

When comparing CFD results for the two fluids with different rheological properties it was also found that for impeller speed *N* ≥6 s^−1^ the non-Newtonian fluid was pulled up from just under the impeller blades, thus in the opposite axial direction than the Newtonian fluid that was pumped down to the tank bottom, what is compatible with literature experimental data (Jaworski and Nienow 1994).

### Strain rate and resulting parameters

From the numerical simulation results, local values of the strain rate, $\dot{\gamma }_{\text{CFD}}$, were also read. The maximum values of the $\dot{\gamma }_{\text{CFD}}$ were achieved close to the impeller blades and significantly decreased with increasing distance from the tank axis. On the basis of the local strain rate values, the other parameters like Metzner–Otto coefficient, *k*_s,CFD_, shear stress, $\tau_{\text{CFD}}$, and intensity of energy dissipation, $\varepsilon_{\text{CFD}}$, can be calculated and analyzed.

**Fig. 13:**
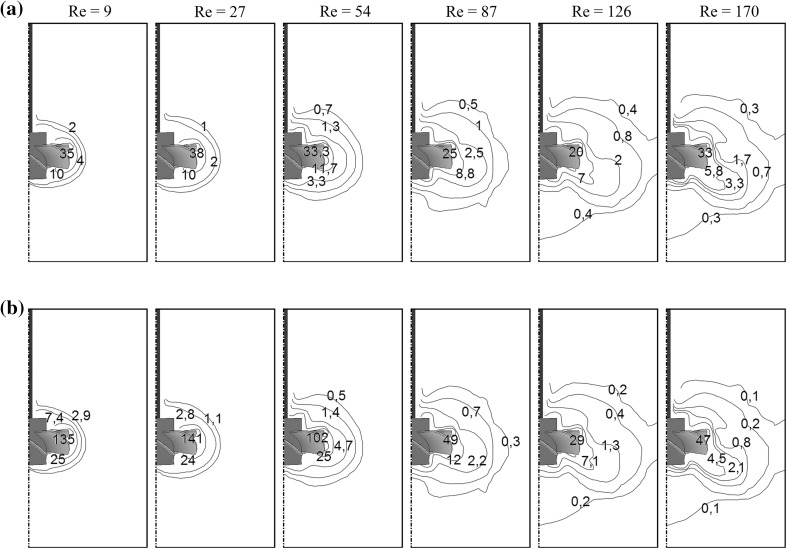
Isoline contours of the local values: **a** Metzner–Otto coefficient, **b** dimensionless intensity of energy dissipation, for *Re* = 9–170, 0.2% Carbopol

Local values of the Metzner–Otto coefficient were calculated from Eq. (18) using an analogy to the Metzner–Otto method (Metzner and Otto 1957):

18 $$k_{\text{s,CFD}} = \frac{{\dot{\gamma }_{\text{CFD}} }}{N}$$

and plotted as isolines in the plane located at the angle of 45° between the baffles (Fig. 13a).

Based on computed local values of *k*_s,CFD_, volumetric mean values of this coefficient, $\bar{k}_{\text{s,CFD}}$, for all volume of the stirred non-Newtonian fluid by the PMT type impeller were calculated in the studied range of the impeller speed. Using the Metzner–Otto method and the $\bar{k}_{\text{s,CFD}}$ values, it was also possible to determine corresponding mean values of the strain rate $\left( {\bar{\dot{\gamma }}_{\text{CFD}} = \bar{k}_{\text{s,CFD}} \cdot N} \right)$ for the whole stirred fluid volume, which were then used in calculating Reynolds numbers at consecutive impeller speeds. The calculated mean values of the $\bar{\dot{\gamma }}_{\text{CFD}}$ and $\bar{k}_{\text{s,CFD}}$ are presented in Table 3.

**Table 3:** Values of the $\bar{\dot{\gamma }}_{\text{CFD}}$, $\bar{k}_{\text{s,CFD}}$ and $\bar{\varepsilon }$ for different values of the *Re* number

*Re*	9	27	54	87	126	170
$\bar{\dot{\gamma }}_{\text{CFD}}$, s^−1^	38	77	119	176	247	319
$\bar{k}_{\text{s,CFD}}$	19	19	20	22	25	27
$\bar{\varepsilon }$, W m^−3^	35	93	189	392	665	1044

It should be underlined that for the studied Reynolds number range, the $\bar{k}_{\text{s,CFD}}$ value varied from 19 to 27. Further analysis of the results revealed that for the nearly laminar fluid flow (*Re*_CFD_ <60) the calculated values of the Metzner–Otto coefficient were similar ($\bar{k}_{\text{s,CFD}}$ = 19 or 20), while for the transitional fluid flow with increasing Reynolds number the value of $\bar{k}_{\text{s,CFD}}$ also increased. Furthermore, CFD simulated value of the $\bar{k}_{\text{s,CFD}}$ was equal to 22 for the impeller speed *N* = 8 s^−1^ (*Re* = 87), whereas literature (Jaworski and Nienow, 1994) quotes for the same impeller rotational speed a similar value of *k*_s_ = 20 that was obtained by means of the Metzner–Otto method and mixing power measurements.

Local values of the shear stress, $\tau_{\text{CFD}}$, were also calculated using Eq. (6). The maximum values of the $\tau_{\text{CFD}}$ were obtained very close to the impeller blades, and they varied from 67 to 122 [Pa] for the studied impeller speed range.

The intensity of energy dissipation, $\varepsilon_{\text{CFD}}$, for the viscous and incompressible fluid obeying the Power Law can be determined from equation:

19 $$\varepsilon_{\text{CFD}} = \tau_{\text{CFD}} \cdot \dot{\gamma }_{\text{CFD}} = K \cdot \dot{\gamma }_{\text{CFD}}^{{\left( {n + 1} \right)}}$$

Dimensionless values of the energy dissipation intensity were then obtained by dividing the local values, $\varepsilon_{\text{CFD}}$, by its mean value, $\bar{\varepsilon }$, calculated from the mixing power and equation $\bar{\varepsilon } = {{4P_{\text{CFD}} } \mathord{\left/ {\vphantom {{4P_{\text{CFD}} } {\left( {\pi T^{2} H} \right)}}} \right. \kern-0pt} {\left( {\pi T^{2} H} \right)}}$. The mean values of the energy dissipation intensity calculated for *Re* = 9–170 are also collected in Table 3. Isolines of the local values of dimensionless intensity of energy dissipation for different *Re* numbers are presented in Fig. 13b. Contours of the local $\varepsilon_{\text{CFD}}$ values indicate for growing *N* a significant increase in size of the zone with high values of $\varepsilon_{\text{CFD}}$, representing the intensive mixing zone.

Similar isoline plots of the local values for velocity, strain rate, Metzner–Otto coefficient, shear stress and intensity of energy dissipation in the impeller swept region (not shown here) also characterized their strong variability, what may be particularly important in stirred bioreactors.

### Cavern diameter

**Fig. 14:**
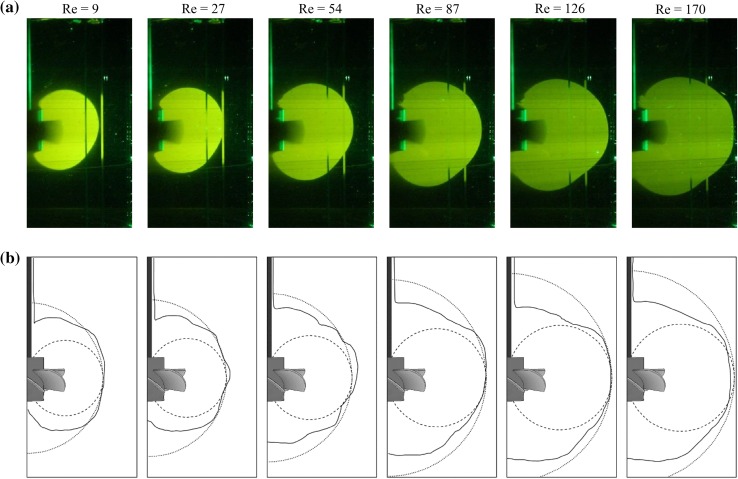
Caverns in the tested system: **a** experiment, **b** CFD (*straight line*), spherical cavern shape (*dotted line*), toroidal cavern shape (*dashed line*)

A cavern boundary for stirred yield stress fluids is usually determined as the place where the shear stress is equal to the fluid yield stress. However, in this paper a viscous fluid without yield stress was considered. For such fluids there is a lack of generally accepted definition of the cavern size. It is not problematic when size of the cavern is determined on the basis of experiment using dye, because the tracer is evenly distributed in the intensive mixing zone. The caverns sizes obtained in this study from experiment are presented in Fig. 14a. The situation is quite different in the case of numerical simulations, where certain assumption should be adopted for cavern size determination. In this study, following the literature (Adams and Barigou 2007), it was assumed that the tangential component of the fluid velocity at the cavern boundary is equal to 1% of the peripheral impeller speed, *v*_TIP_. Very important is also the plane in which the results are read. In this study, the cavern boundaries for simulated tangential velocity of 0.01 *v*_TIP_ were read in the plane located at the angle of 45° between the baffles, thus assuming the maximum possible cavern size. As cavern diameter it was adopted that the maximum horizontal distance between the cavern opposite boundaries projected onto the vertical plane of data reading.

Such cavern boundaries determined from the numerical results for increasing impeller speeds are shown in Fig. 14b as solid lines. In addition, based on the models available in the literature (Amanullah et al. 1998; Adams and Barigou 2007), theoretical spheroidal and toroidal cavern boundaries were also calculated and are shown in Fig. 14b with dotted and dashed lines, respectively.

As it can be seen from Fig. 14, with increasing values of the *Re* number the cavern size also increased, what is consistent with expectations. Cavern diameters obtained from own experiments and numerical simulations and those calculated from the literature models for the same frequency of impeller rotation are shown in Table 4. They were obtained assuming the same fluid velocity as in simulations of 0.01 *v*_TIP_ at the cavern boundary.

**Table 4:** Values of the cavern diameter for different impeller speeds

*N*, s^−1^	2	4	6	8	10	12
*D*_c,exp_, mm	155	164	182	198	211	217
*D*_c,CFD_, mm	158	166	184	200	208	210
*D*_c,lit._, mm^a^	152	158	170	198	210	216

^a^Literature models from Amanullah et al. (1998)

For *N* ≤8 s^−1^, cavern diameters from the numerical simulations are slightly greater than those from the experiment, while for *N* ≥10 s^−1^ they are little smaller. However, the corresponding diameters calculated from the literature models are smaller than those from the experiment for all studied impeller speeds. The results from CFD differed from those of experiment by about 1.6%, while from the literature models by about 2.2%. It should be concluded that both simulation and literature models are good for cavern diameter prediction. It was also observed that none of the predictions describes the cavern shape precisely, however, those calculated from the toroidal equation (dashed line in Fig. 14b) give their shape most similar to the experiment.

In the next step, based on the results of cavern diameter measurements and experimentally determined rheological parameters of the stirred fluid, as well as tangential, *f*_ϕ_, and axial, *f*_ax_, forces, an attempt was made to determine a new model for calculating the diameter of the cavern. First, according to the simplified literature approach (Amanullah et al. 1998), the total force was calculated from both tangential, *f*_ϕ_, and axial, *f*_ax_, forces, as follows:

20 $$F = \sqrt {f_{\phi }^{2} + f_{\text{ax}}^{2} } {\text{ with}}$$

21 $$f_{\phi } = \frac{{4T_{\text{o}} }}{3R}$$

where *T*_o_ is the torque, Nm, *R* is the impeller radius, m.

**Fig. 15:**
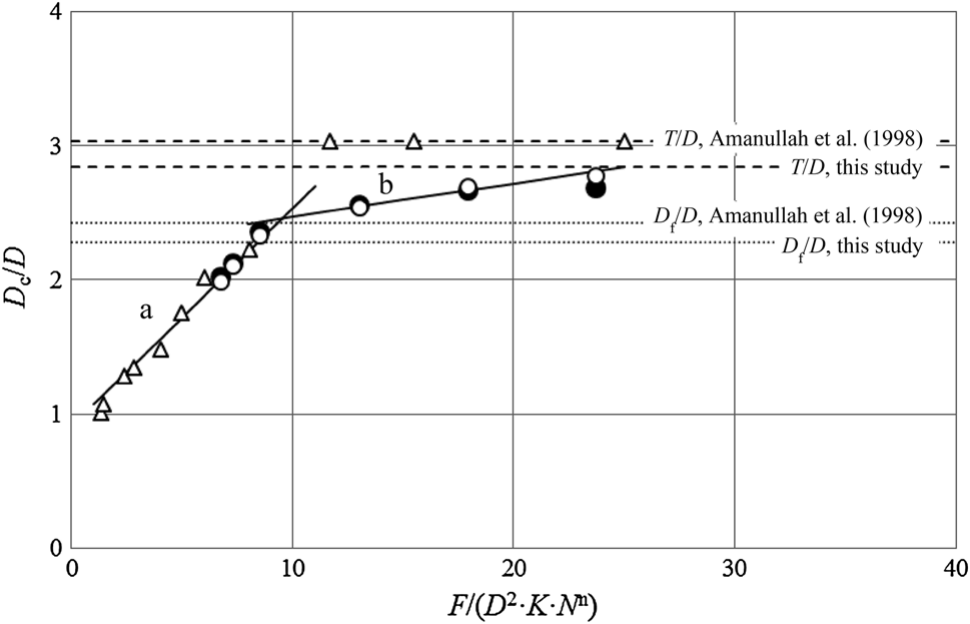
Fitting of the proposed correlations (*line*) to the data from own experiment (*unfilled circle*), numerical simulations (*filled circle*) and experimental data from literature (*triangle*) (Amanullah et al. 1998): **a** Eq. (22a), **b** Eq. (22b)

Second, the cavern diameters, *D*_c_, obtained from measurements were standardized by dividing the impeller diameter, *D*, and then correlated as a two-piece linear function of the newly proposed, dimensionless impeller force number $\left( {\frac{F}{{K \cdot D^{2} \cdot N^{n} }}} \right)$. This is graphically shown in Fig. 15, where also a dashed line is plotted indicating the maximum possible relative cavern size, *T/D*.

An important feature of the standardized cavern diameter, *D*_c_/*D*, obtained both from experiment and CFD, is that its value initially increased rapidly with the force number, and after reaching certain level its further increase was slower. This was attributed to the presence of baffles in the system, i.e. until the cavern reaches the baffles its diameter increases rapidly with increasing impeller speed. However, when the cavern boundary reaches the baffles its increase is slower. Taking into account the above observation, additional correlation line was plotted in Fig. 15 for the cavern diameter beyond the tank baffle limit denoted by *D*_f_ */D* (dotted line, *D*_f_ = *T*−2*w*). It was found that changes in the cavern diameter can be described by two linear equations. The first equation should refer to the cavern diameter in the range *D*_c_ ≤*D*_f_, the second holds within the range *D*_f_ < *D*_c_ ≤ *T*.

The proposed empirical model equations were identified as:

22a $$\frac{{D_{\text{c}} }}{D} = 0.162 \times \left( {\frac{F}{{K \cdot D^{2} \cdot N^{n} }}} \right) + 0.912{\text{ for }}D_{\text{c}} \le D_{\text{f}} = T - 2w$$

22b $$\frac{{D_{\text{c}} }}{D} = 0.025 \times \left( {\frac{F}{{K \cdot D^{2} \cdot N^{n} }}} \right) + 2.22{\text{ for }}D_{\text{f}} < D_{\text{c}} \le T$$

where *D*_c_ is the cavern diameter, m, *D* is the impeller diameter, m, *F* is the total force, *N*, *K* is the fluid consistency coefficient, Pa s^n^, *n* is the flow behavior index, *w* is the baffle width, m. A good fitting of the proposed models to the numerical data is graphically shown in Fig. 15.

To verify that the proposed model equations correctly predict the cavern size in other systems, experimental results from the literature (Amanullah et al. 1998) are additionally shown in Fig. 15. The system studied by Amanullah et al. was equipped with another impeller (SCABA 3SHP1), had other geometric parameters (*D*/*T* = 0.33, *w*/*T* = 0.10) and fluid rheological properties (*K* = 3.8 Pa s^n^, *n* = 0.224). As it can be seen in Fig. 15, for the cavern size *D*_c_ ≤ *D*_f_, the experimental data obtained by Amanullah et al. are very well described by the proposed model (22a) up to *D*_c_ = *T*. Moreover, this model gives a good prediction of cavern diameter even for very low *Re* values, while both literature models—spheroidal and toroidal, were suitable only for *Re* >20. This suggests that the proposed model can be successfully used to predict size of the cavern in various stirred tanks with an axial flow impeller.

## Conclusions

Computational fluid dynamics numerical simulations were successfully performed for a highly shear-thinning fluid flow in a stirred tank equipped with the PMT type impeller, rotating with a constant speed in the range of *N* = 2–12 s^−1^. Based on the simulation results, local values of the velocity, power number, thrust number, shear rate, Metzner–Otto coefficient, shear stress and intensity of energy dissipation in the volume of stirred liquid were calculated.

Validation of the predicted results against the published and own experimental data revealed a good agreement between them. It was found for the 0.2% Carbopol solution and the impeller speed *N* = 8 s^−1^, the values of the mean square deviation for three velocity components were 2.1, 2.5 and 3.6% of *v*_TIP_ for the axial, radial and tangential velocity components, respectively. In the full range of the studied impeller speed, the maximum value of the percentage absolute relative deviation calculated for the power number did not exceed 3.7%, whereas values of the absolute relative deviations calculated from the predicted and experimental values of the dimensionless thrust number ranged from 3.2 to 10.4%.

A similar analysis carried out for water revealed that values of the mean square deviation for three velocity components were 5.0, 3.9 and 1.9% of *v*_TIP_ for the axial, radial and tangential velocity components, respectively. Calculation of the absolute relative deviation for the power number resulted in its value of 2.5%. Thus, it may be concluded that the CFD simulations were favorably validated also for water.

The simulation results showed that stirring of the highly shear-thinning fluid with impeller speed *N* ≥6 s^−1^ produced flow patterns significantly different from those for Newtonian fluids. A large variability of the local values of the velocity, shear rate, Metzner–Otto coefficient, shear stress and intensity of energy dissipation was observed in the intensive mixing zone. Outside this volume, those parameter values were close to zero.

Finally, the new model (Eqs. 22a, 22b) for calculating the cavern diameter of highly shear thinning fluids without a yield stress was proposed and successfully validated on the basis of simulation results and experimental measurements of the cavern diameter, fluid rheological parameters, torque and axial force.
